# Oxidative Stress Related to Iron Metabolism in Relapsing Remitting Multiple Sclerosis Patients With Low Disability

**DOI:** 10.3389/fnins.2019.00086

**Published:** 2019-02-11

**Authors:** Mariacristina Siotto, Maria Maddalena Filippi, Ilaria Simonelli, Doriana Landi, Anna Ghazaryan, Stefano Vollaro, Mariacarla Ventriglia, Patrizio Pasqualetti, Mauro Ciro Antonio Rongioletti, Rosanna Squitti, Fabrizio Vernieri

**Affiliations:** ^1^IRCCS Fondazione Don Carlo Gnocchi, Milan, Italy; ^2^Fatebenefratelli Foundation for Health Research and Education, AFaR Division, Rome, Italy; ^3^Service of Medical Statistics and Information Technology, Fatebenefratelli Foundation for Health Research and Education, AFaR Division, Rome, Italy; ^4^Multiple Sclerosis Clinical and Research Unit, Department of Systems Medicine, Tor Vergata University, Rome, Italy; ^5^Neurology Unit, Campus Bio-Medico University of Rome, Rome, Italy; ^6^Department of Laboratory Medicine, Research and Development Division, “San Giovanni Calibita”, Fatebenefratelli Hospital, Rome, Italy; ^7^IRCCS Istituto Centro San Giovanni di Dio Fatebenefratelli, Brescia, Italy

**Keywords:** multiple sclerosis, oxidative stress, iron metabolism, total antioxidant status, hydroperoxides, ceruloplasmin:transferrin ratio, ceruloplasmin

## Abstract

Oxidative status may play a role in chronic inflammation and neurodegeneration which are considered critical etiopathogenetic factors in Multiple Sclerosis (MS), both in the early phase of the disease and in the progressive one. The aim of this study is to explore oxidative status related to iron metabolism in peripheral blood of stable Relapsing-Remitting MS with low disability. We studied 60 Relapsing-Remitting MS patients (age 37.2 ± 9.06, EDSS median 1.0), and 40 healthy controls (age 40.3 ± 10.86). We measured total hydroperoxides (dROMs test) and Total Antioxidant Status (TAS), along with the iron metabolism biomarkers: Iron (Fe), ferritin (Ferr), transferrin (Tf), transferrin saturation (Tfsat), and ceruloplasmin (Cp) panel biomarkers [concentration (iCp) and enzymatic activity (eCp), copper (Cu), ceruloplasmin specific activity (eCp:iCp), copper to ceruloplasmin ratio (Cu:Cp), non-ceruloplasmin copper (nCp-Cu)]. We computed also the Cp:Tf ratio as an index of oxidative stress related to iron metabolism. We found lower TAS levels in MS patients than in healthy controls (CTRL) and normal reference level and higher dROMs and Cp:Tf ratio in MS than in healthy controls. Cp and Cu were higher in MS while biomarkers of iron metabolism were not different between patients and controls. Both in controls and MS, dROMs correlated with iCp (CTRL *r* = 0.821, *p* < 0.001; MS *r* = 0.775 *p* < 0.001) and eCp (CTRL *r* = 0.734, *p* < 0.001; MS *r* = 0.820 *p* < 0.001). Moreover, only in MS group iCp correlated negatively with Tfsat (*r* = -0.257, *p* = 0.047). Dividing MS patients in “untreated” group and “treated” group, we found a significant difference in Fe values [*F*(2, 97) = 10.136, *p* < 0.001]; in particular “MS untreated” showed higher mean values (mean = 114.5, *SD* = 39.37 μg/dL) than CTRL (mean 78.6, *SD* = 27.55 μg/dL *p* = 0.001) and “MS treated” (mean = 72.4, *SD* = 38.08 μg/dL; *p* < 0.001). Moreover, “MS untreated” showed significantly higher values of Cp:Tf (mean = 10.19, *SD* = 1.77^∗^10^-2^; *p* = 0.015), than CTRL (mean = 9.03, *SD* = 1.46 ^∗^10^-2^). These results suggest that chronic oxidative stress is relevant also in the remitting phase of the disease in patients with low disability and short disease duration. Therefore, treatment with antioxidants may be beneficial also in the early stage of the disease to preserve neuronal reserve.

## Introduction

Multiple sclerosis (MS) is a chronic immune-mediated condition that can affect the brain and the spinal cord and is characterized by a relapsing remitting (RRMS) course eventually followed by secondary progression (SPMS) or gradual progression of disability since the beginning (primary progressive MS – PPMS). MS has been traditionally considered a focal inflammatory demyelinating disease of the white matter, while today the role of chronic and diffuse gray matter neurodegeneration in the disability accrual is well established (Stys et al., 2012). Neurodegeneration accompanies demyelination since the early phases of the disease and becomes the main pathological feature in the secondary and primary progressive forms.

Inflammation and neurodegeneration are mutually dependent phenomena. Inflammation, in fact, induces degeneration, likely through excitotoxicity mechanisms (Centonze et al., 2009); on the other hand neurodegeneration can induce inflammatory response, both in the central nervous system (CNS) and in peripheral blood (Träger and Tabrizi, 2013), as demonstrated also in other neurodegenerative conditions (i.e., amyotrophic lateral sclerosis, Alzheimer’s disease, Parkinson’s disease, and Huntington’s disease) (Träger and Tabrizi, 2013). Nevertheless, consolidated biomarkers of chronic inflammation in MS are lacking, although many studies have identified oxidative stress markers as particularly promising to estimate peripheral inflammation in MS since inflammation leads to oxidative stress and vice-versa (Naidoo and Knapp, 1992; Karg et al., 1999; Besler and Comoǧlu, 2003; Ferretti et al., 2005; Koch et al., 2006; Alimonti et al., 2007; Ortiz et al., 2009, 2013; Ghabaee et al., 2010; Ristori et al., 2011; Miller et al., 2012; Oliveira et al., 2012; Tasset et al., 2012). The majority of these studies have demonstrated an increase of peripheral inflammation in the progressive forms of MS or during a relapse; few of them have suggested that oxidative stress and antioxidant capacity are elevated also in RRMS during the remitting phase (Ferretti et al., 2005; Koch et al., 2006; Miller et al., 2012; Oliveira et al., 2012).

Oxidative status is considered to be related to iron (Fe) metabolism. This metal might play a role in the pathogenesis of inflammation and neurodegeneration in MS, causing microglia activation, induction of mitochondria dysfunction and free radicals in the body and in the CNS (Drayer et al., 1987; Bizzi et al., 1990; Zivadinov et al., 2010, 2018; Sheykhansari et al., 2018). Ceruloplasmin (Cp) is an acute phase protein (Hellman and Gitlin, 2002), playing a fundamental role in copper (Cu) and Fe metabolism and holding a strong antioxidant function due to its ferroxidase activity (Gutteridge, 1995): indeed, the Cp:Transferrin (Tf) system (measured by the Cp:Tf ratio) is reported to be the main antioxidant system in peripheral blood (Kozlov et al., 1984). Cp has also been recognized as marker of inflammation in systemic pathologies (Göçmen et al., 2008; Tang et al., 2012) and we found that the Cp:Tf ratio is elevated in Alzheimer’s disease (Squitti et al., 2010; Siotto et al., 2016), in stroke (Altamura et al., 2009; Squitti et al., 2018b) and in subacute post-stroke patients affected by neuropathic pain (Siotto et al., 2017), where it correlates with the clinical status.

Our study aimed to identify possible and easy to test markers of peripheral inflammation and to explore Fe metabolism in relation to the clinical status in MS patients with low disability in the remitting phase. For this purpose, we used two commercially available biomarker of oxidative stress, along with a panel of biomarkers related to Fe metabolism, strictly associated with oxidative stress.

## Materials and Methods

### Subjects

The study was performed at Neuroscience Department of the Fatebenefratelli Hospital, Isola Tiberina, Rome and at Neurology Unit of the Campus Bio-Medico University of Rome.

Sixty Relapsing-Remitting MS patients (45 females, age 37.2 ± 9.06) fulfilling the 2010 revision of diagnostic criteria of MS (Polman et al., 2011) were recruited. Forty healthy unrelated volunteers (22 females, age 40.3 ± 10.86) of comparable age were also selected as control group (CTRL). All the included patients were free from relapses for at least 6 months before the blood sampling.

Exclusion criteria were the following: therapy with corticosteroids or ACTH in the month before the blood sampling, pregnancy, anemia, alcohol, and drug abuse, use of dietary supplements, chronic diseases potentially inducing systemic inflammation (heart or pulmonary diseases, diabetes, autoimmune diseases, etc.), primary or secondary hepatic diseases, hemochromatosis, aceruloplasminemia and any other diseases with known or presumable effect on Cu/Fe metabolism.

Local institutional ethics committees approved the study and all participating subjects gave written informed consent to be included in the study, in line with the Code of Ethics of the World Medical Association (Declaration of Helsinki) and the standards established by the Authors’ Institutional Review Board.

### Blood Sampling and Biochemical Assay

Fasting blood samples were collected in the morning and sera fractions were separated by centrifugation (3000 rpm, 10 min, and 4°C). They were then aliquoted and rapidly stored at -80°C. The subject’s aliquots and the reference samples were thawed just before the assay.

All the serum analyses were performed in duplicate on the biochemical analyzer Horiba Pentra 400 (ABX Diagnostic, Montpellier, France).

Total antioxidant blood capacity (TAS) was measured using the TAS kit (Randox Laboratories, Crumlin, United Kingdom) (Rice-Evans and Miller, 1994). Hydroperoxide content was assessed by d-ROMs test (Diacron, Italy) and expressed in arbitrary units (U.CARR) where 1 U.CARR corresponding to 0.08 mg/100 ml of hydrogen peroxide (Alberti et al., 2000).

Iron (Fe) was measured by the photometric test using Ferene (Higgins, 1981) (Horiba ABX, Montpellier, France). Transferrin (Tf) levels were measured by immunoturbidimetric assay (Skikne et al., 1990) and Ferritin was measured by latex-enhanced turbidimetric immunoassay (Simó et al., 1994) (Horiba ABX, Montpellier, France). Concentration of immunological Cp (iCp) was measured by immunoturbidimetric assay (Wolf, 1982) (Futura System SRL, Rome, Italy). The enzymatic activity of Cp (eCp) was tested with an automated version of the manual Schosinsky *o*-dianisidine eCp assay (Lehmann et al., 1974; Schosinsky et al., 1974), adapted by our laboratory (Siotto et al., 2014). The serum copper (Cu) concentration was measured using the colorimetric assay of Abe et al. (1989) (Randox Laboratories, Crumlin, United Kingdom).

For each sample, we also computed the specific activity of Cp (enzymatic activity *per* mg of Cp concentration in IU/mg^∗^10^-1^), as the ratio between the Cp enzymatic activity and immunoturbidimetric Cp concentration (eCp/iCp) (Siotto et al., 2014) and the ratio between Cp and Tf serum concentrations (Cp:Tf ^∗^10^-2^) (Nobili et al., 2013). nCp-Cu was calculated by means of the equation provided by Walshe [appendix of Walshe (2003)] on the basis of total copper and iCp concentrations in serum. For each serum copper and ceruloplasmin pair, we computed the amount of copper bound to ceruloplasmin (CB) and the amount of nCp-Cu, following standard procedures (Appendix 1: “Calculation of ‘free copper’ concentration”). The Cu:Cp ratio (Twomey et al., 2006; Squitti et al., 2014) was calculated as reported in Twomey et al. (2006). Moreover, Tf saturation (Tf-sat) was calculated by dividing serum Fe (μg/dL) by the total iron binding capacity (TBC = TF in mg/dL^∗^1.25) and multiplying by 100.

### Statistical Analysis

Smirnov test was applied to test the normality distribution of continuous variables. To test difference in age and clinical characteristics between two groups was applied the Student’s *t*-test or, when necessary, non-parametric Mann-Whitney test. A logarithmic transformation was applied to minimize the variability and to better approximate the data distribution to normality.

Correlation between all biochemical variables were calculated by the Pearson’s correlation coefficient separately in the MS and CTRL groups to look for any differences in the reciprocal behavior of the variables between the two groups. Non-parametric Spearman’s correlation was calculated to test the correlation between the biochemical variables and the clinical variables (EDSS, disease duration). Analysis of variance (ANOVA) model was applied to evaluate the difference in the biochemical variables between CTRL and MS patients and between CTRL and MS divided in “untreated MS” and “treated MS,” adjusting for sex and age. The results were presented as mean and standard deviation (*SD*). Benjamini-Hochberg procedure was applied to adjust the *p*-value in multiple comparisons. A *p*-value < 0.05 has been considered statistically significant. The statistical analysis was performed using IBM SPSS Statistics for Windows version 19.0.0.

## Results

All patients had a low disability [Expanded Disability Status Scale, EDSS 1.0 (0.0–4.5)], except for a single male patient with EDSS 4.5. Patients on disease modifying therapy (DMT) were 78%, of which 85% treated with interferon-beta (Significant demographic and clinical data are listed in Table 1). Patients and controls did not diverge for age but were different for sex distribution (*p* = 0.018, see Table 1).

**Table 1:** Demographic of multiple sclerosis patients (MS) and healthy volunteers (CTRL).

	*n*	Age Mean (*SD*)	Disease duration Median (range)	EDSS Median (IQR)	Patients on Disease Modifying Therapy (DMT) (*n*)
**MS patients**	**60**	**37.2 (9.06)**	**2.39 (0.01–19.86)**	**1.0 (1**–**2)**	**41 (78%)**
Females	45	36.0 (9.08)	3.9 (0.2–20.1)	1.0 (0.0–1.75)	32
**Controls**	**42**	**40.3 (10.86)**			
Females	22	40.4 (11.05)			


*In non-bold are reported data of Female MS and Female CTRL.*

**FIGURE 1:**
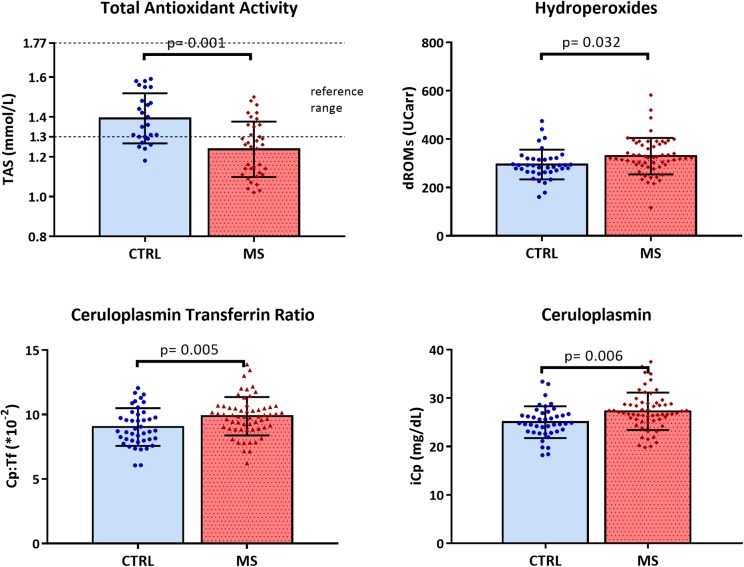
Scattered dot plot with bar plot (mean with *SD*) of total antioxidant status (TAS), hydroperoxides (dROMs), ceruloplasmin transferrin ratio (Cp:Tf), and ceruloplasmin (iCp) in CTRL and MS.

Differences in biochemical variables, adjusted for sex and age in ANOVA analyses, between MS patients and CTRL were reported in Table 2. TAS was significantly lower in patients than in controls (MS mean = 1.24, *SD* = 0.14 vs. CTRL mean = 1.39, *SD* = 0.13 mmol/L; *p* = 0.001) and lower than normal reference range indicated by the manufacturer (1.30–1.77 mmol/L). The total hydroperoxides in circulation were higher in MS patients than in healthy volunteers (MS mean = 329.6, *SD* = 75.15 vs. CTRL mean = 295.2, *SD* = 61.38 UCarr; *p* = 0.032). Moreover, the Cp:Tf ratio was higher in MS patients than in controls (MS mean = 9.89, *SD* = 1.48^∗^10^-2^ vs. CTRL mean = 9.03, *SD* = 1.46 ^∗^10^-2^; *p* = 0.005) (Table 2 and Figure 1).

**Table 2:** Biological variable differences in multiple sclerosis patients (MS) and in healthy volunteers (CTRL).

Biochemical variables		MS *n* = 60	CTRL *n* = 42	ANOVA^a^ *p*-value
Total antioxidant capacity (TAS, mmol/L)	Mean (*SD*)	1.24 (0.14)	1.39 (0.13)	**0.001**
Hydroperoxides, dROMs (UCarr)	Mean (*SD*)	329.6 (75.15)	295.2 (61.38)	**0.032**
Iron (Fe, μg/dL)	Mean (*SD*)	85.7 (42.97)	78.6 (27.55)	0.268
Ferritin (Ferr, ng/mL)	Median (25–75)	42.2 (22.8–110.17)	39.6 (26.23–112.47)	0.236^b^
Transferrin (Tf, g/L)	Mean (*SD*)	2.75 (0.05)	2.79 (0.05)	0.616
Ceruloplasmin (iCp, mg/dL)	Mean (*SD*)	26.9 (3.86)	24.8 (3.28)	**0.006**
Copper (Cu, μmol/L)	Mean (*SD*)	13.74 (0.29)	12.84 (0.33)	**0.043**
Ceruloplasmin activity (eCp, IU/L)	Mean (*SD*)	105.5 (21.94)	97.6 (19.14)	0.142
Cp:Tf ratio (^∗^10^-2^)	Mean (*SD*)	9.89 (1.485)	9.03 (1.463)	**0.005**
Transferrin saturation (Tf sat, %)	Mean (*SD*)	25.2 (13.96)	22.7 (8.43)	0.192
Ceruloplasmin specific activity (eCp/iCp IU/mg^∗^10^-1^)	Mean (*SD*)	3.85 (0.09)	3.91 (0.10)	0.667
Copper not bound to ceruloplasmin (nCp-Cu, μmol/L)	Mean (*SD*)	1.05 (0.2)	1.12 (0.22)	0.832
Cu:Cp ratio	Mean (*SD*)	6.76 (0.11)	6.85 (0.12)	0.589


*^*a*^ANOVA model was applied to evaluated the difference in the biochemical variables between healthy controls and SM patients adjusting for sex and age.*

*^*b*^ANOVA model was applied considered the logarithmic transformation of the variable.*

*Bold indicates *p* < 0.05.*

The iCp values were higher in patients with respect to healthy controls (MS mean = 26.9, *SD* = 3.86 vs. CTRL mean = 24.8, *SD* = 3.28 mg/dL; *p* = 0.006) and coherently the Cu values were a slightly higher in patients (MS mean = 13.7, *SD* = 0.29 vs. CTRL mean = 12.8, *SD* = 0.33 μmol/L; *p* = 0.043; Figure 1).

**FIGURE 2:**
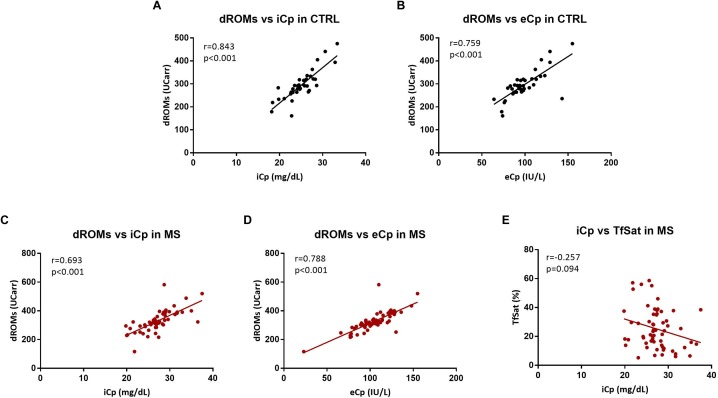
**(A,B)** Correlation between hydroperoxides (dROMs) and ceruloplasmin (iCp and eCp, respectively) in CTRL. **(C,D)** Correlation between hydroperoxides (dROMs) and ceruloplasmin (iCp and eCp, respectively) in MS. **(E)** Correlation between ceruloplasmin (iCp) vs. transferrin saturation (TfSat %) in MS. A *p*-value < 0.05 was considered statistically significant.

The following biological variables correlated both in healthy controls and MS patients: dROMs with iCp (CTRL: *r* = 0.821, *p* < 0.001; MS: *r* = 0.775 *p* < 0.001) and eCp (CTRL: *r* = 0.734, *p* < 0.001; MS: *r* = 0.820 *p* < 0.001). Moreover, in MS patients iCp correlated negatively with Tfsat (*r* = -0.257, *p* = 0.047) but it not survived after the adjustment for multiple comparisons (B-H adjusted *p* = 0.094) (Figure 2). No additional correlations were found among any of the other biochemical parameters and EDSS, disease duration (all *p* > 0.2).

Considering untreated MS patients and treated MS as two separate groups, we did not observe differences in disease duration (Mann Whitney test *p* = 0.10) and EDSS (Mann Whitney test *p* = 0.448).

**FIGURE 3:**
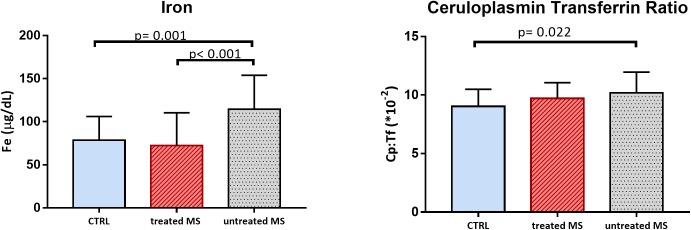
Bar plot (mean with *SD*) of iron (Fe) and ceruloplasmin transferrin ratio (Cp:Tf) in CTRL, MS in disease modifying therapy (treated MS), and in MS not in disease modifying therapy (untreated MS).

We performed the ANOVA analyses comparing the two groups of MS patients with CTRL. All analyses were adjusted for sex and age. TAS was significantly lower in both patients’ groups than in controls (“untreated MS” mean = 1.25, *SD* = 0.14 mmol/L and “treated MS” mean = 1.23, *SD* = 0.15 mmol/L vs. CTRL mean = 1.39 *SD* = 0.15 mmol/L; *p* = 0.015 and *p* = 0.005, respectively) which is consistent with our previous observations. The total hydroperoxides in circulation were no longer significantly different between the three groups (*p* = 0.081). Dividing MS patients in “untreated” group and “treated” group, we found a significant difference in Fe values [*F*(2, 97) = 10.136, *p* < 0.001]; in particular “untreated MS” showed higher mean values (mean = 114.5, *SD* = 39.37 μg/dL) than CTRL (mean 78.6, *SD* = 27.55 μg/dL *p* = 0.001) and “treated MS” (mean = 72.4, *SD* = 38.08 μg/dL; *p* < 0.001). Moreover, “untreated MS” presented significantly higher values of Cp:Tf (mean = 10.19, *SD* = 1.77^∗^10^-2^; *p* = 0.015), than CTRL (mean = 9.03, *SD* = 1.46 ^∗^10^-2^) (Figure 3).

## Discussion

The main result of this study is that in RRMS patients with low disability in the early phase of disease, the oxidative stress status is altered, as it is revealed by low levels of TAS, and high levels of total hydroperoxides and of Cp:Tf (Table 2 and Figure 1). In fact, even though EDSS scores were low, suggesting a low rate of neurodegeneration, all the serum biomarkers of oxidative status analyzed revealed a marked imbalance in systemic oxidative stress.

Another interesting result is that dividing MS patients in “treated” and “untreated,” we found increased level of Fe and Cp:Tf ratio in “MS untreated,” suggesting a role of therapy in oxidative stress related to Fe levels (Figure 3).

We employed TAS and dROMs as oxidative stress markers, two easy to use and common laboratory assays to test general oxidative stress status of the body, and the Cp: Tf ratio which is an index optimized from our laboratory to explore oxidative stress mediated by Fe.

The TAS is a measure of the total antioxidant capacity of general circulation. The biochemical assay is based on the principle of inducing the antioxidant defense of serum; this is achieved by introducing a radical reagent opportunely and previously formed in the sample; the serum sample is then added. Serum can exert a suppression of the radical in a way that is directly proportional to the ability of all antioxidant components in serum to counteract the radical oxidation. We found that serum samples of MS patients were not able to counteract the induced oxidative stress, as revealed by TAS values lower than healthy control and reference range (Table 2 and Figure 1). No differences in TAS levels were found between “treated MS” and “untreated MS”; TAS values were significantly lower in both patients’ group and under the reference range.

The dROMs is a test that measures directly the hydroperoxides circulating in serum (Cesarone et al., 1999; Alberti et al., 2000). High dROMs have been found in patients with different chronic conditions, correlating with the clinical outcome (Daniil et al., 2008; Capone et al., 2012; Vassalle et al., 2012; Taguchi et al., 2013) and with C reactive protein (Kotani and Taniguchi, 2012; Taguchi et al., 2013). Moreover, antioxidant administration has been demonstrated to reduce dROMs (Cornelli et al., 2001). In this study we noticed a very strong positive correlation between iCp and eCp and dROMs (both in healthy CTRL and in MS with a Spearman’s rho close to 0.8, Figure 2). On this basis, dROMs test appears as an alternative measurement of Cp enzymatic activity, or related to the antioxidant activity of Cp. Erel et al. (Harma, 2006) sustained that the chromogen employed in dROMs test was a substrate of the ferroxidase Cp while another study asserted that there is no correlation between ceruloplasmin and dROMs (Colombini et al., 2016). Nevertheless, the hypothesis that enzymatic *o*-dianisidine assays such as *e*Cp might in turn be influenced by the presence of blood hydroperoxides cannot be excluded. Whether measuring oxidative stress or the ferroxidase activity of Cp (as sustained by Erel), dROMs is a reliable method to detect a peripheral response to a systemic stress.

In this study we found dROMs levels higher in MS compared to healthy controls (Table 2 and Figure 1) but no differences were found dividing patients in treated or not.

In order to further explore the oxidative stress status in MS, we also measured the Cp:Tf ratio and we found higher values in MS than in CTRL (Table 1 and Figure 1) and in the group of “untreated MS” (Figure 3). This ratio is reported to be the main antioxidant system in serum, and it was measured with Electron Paramagnetic Resonance Spectroscopy by Kozlov et al. (1984). The Cp:Tf variable analyzed in this study, is a calculated index that provides a good quantification of this antioxidant system (Altamura et al., 2009; Nobili et al., 2013). In fact, it quantifies the existing ratio between Cp and Tf. A correct “handling” and distribution of Fe is evinced by a correct stoichiometry between these two proteins. When this occurs, there is consequently a lower probability to develop oxidative stress because the two proteins work in concert reducing levels of ferrous ions, Fe^2+^, the Fe ion more toxic (Singh et al., 2012) that reacts with hydrogen peroxides initiating oxidative stress chain reactions via Fenton’s and Heber Weiss reactions and generating reactive oxygen species.

According to the hypothesis of cerebrospinal venous insufficiency (Zamboni et al., 2009; Zivadinov et al., 2010) abnormality in blood drainage from the brain and spinal cord may contribute to nervous system damage in MS through Fe overload and deposition (Singh and Zamboni, 2009; Singh, 2010). In 2018 a clinical trial study showed no efficacy of venous percutaneous transluminal angioplasty (Zamboni et al., 2018); in fact, the treatment did not increase the proportion of patients who improved functionally nor did it reduce the mean number of new combined brain lesions on magnetic resonance imaging (MRI) at 12 months. However, the CCSVI hypothesis has been linked with the potential effects of Fe deposition in the brain parenchyma. Together with radiological data which suggest excessive Fe accumulation in the brain of MS patients (Drayer et al., 1987; Bizzi et al., 1990; Zivadinov et al., 2010), this hypothesis drove researchers attention upon the Fe as a possible cause of neurodegeneration, although the reasons of Fe overload are not clear yet and presumably they do not depend on CCSVI. The brain Fe accumulation could lead to chronic cell stress, with consequent axonal and neuronal death. Moreover, there are some heme-involved processes that caused lipid oxidation. During de-myelinization, Fe could be released from myelin sheath (for an extensive review see Adamczyk and Adamczyk-Sowa, 2016).

However, we did not find any significant difference between MS group and controls in the markers strictly related to Fe as Fe itself, Ferr, and Tf. On the other hand, we found significant higher level of Fe in “untreated MS” respect to both control and “treated MS” (Table 2 and Figure 3). Despite preliminary and limited by the small sample size, these results are quite interesting as they highlight the importance of better investigating the role of treatment in Fe metabolism.

Looking at the other variables, we also found iCp and Cu higher in MS patients compared to healthy controls. Cp is acute-phase reactant in inflammation processes (such as infection, chronic diseases, arthritis and several neoplasia) and Cu follows the same trend being structurally bound to Cp. Holo-Cp biosynthesis occurs in liver where the Cu is charged in the apo-form of the protein. The Cu in the Cp is not exchangeable, being part of the protein structure. So, in MS, the rise of Cp can explain also the rise of Cu due to an inflammation status. Recently, a study from De Riccardis et al. (2018) on 38 MS patients with EDSS scores under 3.5 reported that Cp and Cu values were higher in MS than in controls of an age and sex matched group of 39 subjects, according to our results. Moreover, they found a significant increase in dROMs values in MS patients.

In our study we found no significative difference in other important markers of Cu status like nCp-Cu that measures the portion of Cu not structurally bound to Cp, and Cu:Cp ratio which is an index of the correct stoichiometry between Cu and Cp (Table 2). Conversely, in various study on different neurological diseases, such as Alzheimer’s or Wilson’s disease (Siotto et al., 2016; Squitti et al., 2018a) or stroke (Siotto et al., 2017; Squitti et al., 2018b) we found these specific markers of Cu status altered, with a clear indication of Cu dishomeostasis.

In our opinion, in our MS group iCp and Cu were higher due to systemic inflammation and not as a result of an imbalance systemic Cu management.

Cp is involved in the metabolism of Fe being the main ferroxidase of the serum, as it oxidizes Fe from Fe^2+^ to Fe^3+^, which could be charged from Tf for being transported into the circulation. The increase of iCp in MS and its negative correlation with TfSat stands for an impairment in the Fe handling, being that in MS the more circulating Cp correlates with less TfSat (Letendre and Holbein, 1984; Hellman and Gitlin, 2002). Transferrin itself showed no differences between MS and CTRL as reported also in a study on CSF samples (LeVine et al., 1999).

Summarizing our results, the oxidative stress is clearly an evidence in MS patients, but while the values of TAS and dROMs appeared not to be influenced by therapy, underlining the general systemic oxidative stress in MS, Cp:Tf ratio is a good index to detect oxidative stress strictly related to Fe and potentially related with therapy.

Cp:Tf ratio values is an easy and good marker to investigate the mismanagement of Fe. A higher Cp:Tf ratio has been observed in two studies on stroke patients during acute phase (Altamura et al., 2009; Squitti et al., 2018b). Moreover, we recently found an increase of Cp:Tf ratio in subacute stroke patients affected by neuropathic pain (Siotto et al., 2017). In this group of patients, TAS levels were lower than normal, whereas the Cp:Tf ratio was higher. Decreased TAS levels indicated depletion of the antioxidant system compounds and a reduced ability to counteract the increased oxidative stress generated by stroke injury. The parallel increase of Cp:Tf ratio revealed the activation of systemic processes leading to the cellular internalization of Fe in order to contain oxidative stress in subacute post-stroke patients with neuropathic pain (Carbonell and Rama, 2007).

Similarly, TAS levels, being lower in our group of MS patients, than both in healthy controls and in the reference range, reveal an inability to counteract oxidative stress in patients with early MS. In the meantime, the Cp:Tf system responds to a general status of oxidation (as depicted also from higher levels of hydroperoxides) and is activated to exert a cellular internalization of Fe, especially in patients with higher levels of Fe.

In a study by Oliveira et al. (2012) oxidative stress in MS was evaluated by measuring various oxidative biomarkers. Compared to controls, MS patients with EDSS > 3.5 exhibited higher plasma levels of tert-butyl hydroperoxide-initiated chemiluminescence and carbonyl protein, as well as lower plasma levels of nitric oxide, total radical-trapping antioxidant parameter (measured by the same test we used in current study). In agreement with our results, Oliveira et al. (2012) demonstrated that oxidative stress is important in the physiopathology of MS progression. Is it well known that reactive oxygen species are generated in excess by macrophages which have been implicated as mediators of demyelination and axonal damage in both MS and experimental autoimmune animal models (Gilgun-Sherki et al., 2004).

Another study exploring the value of oxidative stress biomarkers in MS patients reported that plasmatic advanced oxidation protein products were significantly higher while ferric reducing ability and thiol group levels were lower in MS patients in comparison with healthy controls (Pasquali et al., 2015).

Our results underline the importance to monitor oxidative stress and Fe in patients, both at the beginning of the disease and during the DMT. The blood tests employed in this study are easier to perform and cheaper than other serum and radiological biomarkers. MRI is a very well-established method for qualitative and quantitative assessment of MS related damage, but it needs clinical expertise and cumbersome instrumentation set up.

## Conclusion

An altered status of oxidative stress and/or antioxidant response is detectable in MS patients with low disability analyzed during a relapse-free period, suggesting the occurrence of a systemic subclinical inflammation that accompanies MS even in the early stages and in the remitting phase. Assessment of the oxidative stress biomarkers and particularly of Cp:Tf ratio, which is strictly related to Fe management, is an easy way to monitor oxidative stress in MS. Moreover, adding antioxidants to conventional immunotherapy in MS may be reasonable and highly beneficial for MS patients due to their ability to reduce oxidative stress. Further research should be performed to test new antioxidants, and to develop new methods to monitor oxidative stress.

## Author Contributions

MMF, DL, AG, SV, and FV contributed to patient enrolment and clinical evaluation. MS, RS, and MV contributed to blood sample collection and biochemical evaluation. MS, IS, MMF, PP, MCAR, RS, and FV contributed to study design, statistical analysis, data interpretation, and manuscript preparation.

## Conflict of Interest Statement

SR is the inventor of a Cu-related kit for AD diagnosis and minor shareholder in IGEA Research Co (3% shares) and in Canox4Drug SPA (3%) without monetary compensation. The remaining authors declare that the research was conducted in the absence of any commercial or financial relationships that could be construed as a potential conflict of interest.

## References

[B1] Abe A. Yamashita S. Noma A. (1989). Sensitive, direct colorimetric assay for copper in serum. *Clin. Chem.* 35 552–554. 2702740

[B2] Adamczyk B. Adamczyk-Sowa M. (2016). New insights into the role of oxidative stress mechanisms in the pathophysiology and treatment of multiple sclerosis. *Oxid. Med. Cell. Longev.* 2016:1973834. 10.1155/2016/1973834 27829982 PMC5088319

[B3] Alberti A. Bolognini L. Macciantelli D. Caratelli M. (2000). The radical cation of N,N-Diethyl-para-phenylendiamine: a possible indicator of oxidative stress in biological samples. *Res. Chem. Intermed.* 26 253–267. 10.1163/156856700X00769

[B4] Alimonti A. Ristori G. Giubilei F. Stazi M. A. Pino A. Visconti A. (2007). Serum chemical elements and oxidative status in Alzheimer’s disease, Parkinson disease and multiple sclerosis. *Neurotoxicology* 28 450–456. 10.1016/j.neuro.2006.12.001 17267042

[B5] Altamura C. Squitti R. Pasqualetti P. Gaudino C. Palazzo P. Tibuzzi F. (2009). Ceruloplasmin/Transferrin system is related to clinical status in acute stroke. *Stroke* 40 1282–1288. 10.1161/STROKEAHA.108.536714 19228837

[B6] Besler H. T. Comoǧlu S. (2003). Lipoprotein oxidation, plasma total antioxidant capacity and homocysteine level in patients with multiple sclerosis. *Nutr. Neurosci.* 6 189–196. 10.1080/1028415031000115945 12793524

[B7] Bizzi A. Brooks R. A. Brunetti A. Hill J. M. Alger J. R. Miletich R. S. (1990). Role of iron and ferritin in MR imaging of the brain: a study in primates at different field strengths. *Radiology* 177 59–65. 10.1148/radiology.177.1.2399339 2399339

[B8] Capone F. Guerriero E. Sorice A. Maio P. Colonna G. Castello G. (2012). Characterization of metalloproteinases, oxidative status and inflammation levels in the different stages of fibrosis in HCV patients. *Clin. Biochem.* 45 525–529. 10.1016/j.clinbiochem.2012.02.004 22366372

[B9] Carbonell T. Rama R. (2007). Iron, oxidative stress and early neurological deterioration in ischemic stroke. *Curr. Med. Chem.* 14 857–874. 10.2174/092986707780363014 17430141

[B10] Centonze D. Muzio L. Rossi S. Furlan R. Bernardi G. Martino G. (2009). The link between inflammation, synaptic transmission and neurodegeneration in multiple sclerosis. *Cell Death Differ.* 17 1083–1091. 10.1038/cdd.2009.179 19927157

[B11] Cesarone M. R. Belcaro G. Carratelli M. Cornelli U. De Sanctis M. T. Incandela L. (1999). A simple test to monitor oxidative stress. *Int. Angiol.* 18 127–130. 10424368

[B12] Colombini F. Carratelli M. Alberti A. (2016). Oxidative stress, d-ROMs test, and ceruloplasmin. *Free Radic. Res.* 50 447–453. 10.3109/10715762.2015.1136063 26729560

[B13] Cornelli U. Terranova R. Luca S. Cornelli M. Alberti A. (2001). Bioavailability and antioxidant activity of some food supplements in men and women using the d-Roms test as a marker of oxidative stress. *J. Nutr.* 131 3208–3211. 10.1093/jn/131.12.3208 11739867

[B14] Daniil Z. D. Papageorgiou E. Koutsokera A. Kostikas K. Kiropoulos T. Papaioannou A. I. (2008). Serum levels of oxidative stress as a marker of disease severity in idiopathic pulmonary fibrosis. *Pulm. Pharmacol. Ther.* 21 26–31. 10.1016/j.pupt.2006.10.005 17161968

[B15] De Riccardis L. Buccolieri A. Muci M. Pitotti E. De Robertis F. Trianni G. (2018). Copper and ceruloplasmin dyshomeostasis in serum and cerebrospinal fluid of multiple sclerosis subjects. *Biochim. Biophys. Acta Mol. Basis Dis.* 1864(5 Pt A), 1828–1838. 10.1016/j.bbadis.2018.03.007 29524632

[B16] Drayer B. Burger P. Hurwitz B. Dawson D. Cain J. (1987). Reduced signal intensity on MR images of thalamus and putamen in multiple sclerosis: increased iron content? *AJR Am. J. Roentgenol.* 149 357–363. 10.2214/ajr.149.2.357 3496764

[B17] Ferretti G. Bacchetti T. Principi F. Di Ludovico F. Viti B. Angeleri V. A. (2005). Increased levels of lipid hydroperoxides in plasma of patients with multiple sclerosis: a relationship with paraoxonase activity. *Mult. Scler.* 11 677–682. 10.1191/1352458505ms1240oa 16320727

[B18] Ghabaee M. Jabedari B. Al-E-Eshagh N. Ghaffarpour M. Asadi F. (2010). Serum and cerebrospinal fluid antioxidant activity and lipid peroxidation in guillain-barre syndrome and multiple sclerosis patients. *Int. J. Neurosci.* 120 301–304. 10.3109/00207451003695690 20374079

[B19] Gilgun-Sherki Y. Melamed E. Offen D. (2004). The role of oxidative stress in the pathogenesis of multiple sclerosis: the need for effective antioxidant therapy. *J. Neurol.* 251 261–268. 10.1007/s00415-004-0348-9 15015004

[B20] Göçmen A. Y. Sahin E. Semiz E. Gümuşlü S. (2008). Is elevated serum ceruloplasmin level associated with increased risk of coronary artery disease? *Can. J. Cardiol.* 24 209–212. 18340391 10.1016/s0828-282x(08)70586-5 PMC2649635

[B21] Gutteridge J. M. C. (1995). Lipid peroxidation and antioxidants as biomarkers of tissue damage. *Clin. Chem.* 41 1819–1828. 7497639

[B22] Harma M. I. (2006). d-ROMs Test Detects Ceruloplasmin, Not Oxidative Stress. *CHEST J.* 130:1276. 10.1378/chest.130.4.1276 17035469

[B23] Hellman N. E. Gitlin J. D. (2002). Ceruloplasmin metabolism and function. *Annu. Rev. Nutr.* 22 439–458. 10.1146/annurev.nutr.22.012502.114457 12055353

[B24] Higgins T. (1981). Novel chromogen for serum iron determinations. *Clin. Chem.* 27 1619–1620. 7261343

[B25] Karg E. Klivényi P. Németh I. Bencsik K. Pintér S. Vécsei L. (1999). Nonenzymatic antioxidants of blood in multiple sclerosis. *J. Neurol.* 246 533–539. 10.1007/s004150050399 10463352

[B26] Koch M. Ramsaransing G. S. M. Arutjunyan A. V. Stepanov M. Teelken A. Heersema D. J. (2006). Oxidative stress in serum and peripheral blood leukocytes in patients with different disease courses of multiple sclerosis. *J. Neurol.* 253 483–487. 10.1007/s00415-005-0037-3 16283096

[B27] Kotani K. Taniguchi N. (2012). Correlation between high-sensitivity C-reactive protein and reactive oxygen metabolites during a one-year period among asymptomatic subjects. *J. Clin. Med. Res.* 4 52–55. 10.4021/jocmr755w 22383928 PMC3279502

[B28] Kozlov A. V. Sergienko V. I. Vladimirov Iu. A Azizova O. A. (1984). The antioxidant system of transferrin-ceruloplasmin in experimental hypercholesterolemia. *Biull. Eksp. Biol. Med.* 98 668–671. 6095951

[B29] Lehmann H. P. Schosinsky K. H. Beeler M. F. (1974). Standardization of serum ceruloplasmin concentrations in international enzyme units with o dianisidine dihydrochloride as substrate. *Clin. Chem.* 20 1564–1567. 4430134

[B30] Letendre E. D. Holbein B. E. (1984). Ceruloplasmin and regulation of transferrin iron during *Neisseria meningitidis* infection in mice. *Infect. Immun.* 45 133–138. 6429041 10.1128/iai.45.1.133-138.1984 PMC263289

[B31] LeVine S. M. Lynch S. G. Ou C. N. Wulser M. J. Tam E. Boo N. (1999). Ferritin, transferrin and iron concentrations in the cerebrospinal fluid of multiple sclerosis patients. *Brain Res.* 821 511–515. 10.1016/S0006-8993(98)01360-2 10064838

[B32] Miller E. Walczak A. Saluk J. Ponczek M. B. Majsterek I. (2012). Oxidative modification of patient’s plasma proteins and its role in pathogenesis of multiple sclerosis. *Clin. Biochem.* 45 26–30. 10.1016/j.clinbiochem.2011.09.021 22019955

[B33] Naidoo R. Knapp M. L. (1992). Studies of lipid peroxidation products in cerebrospinal fluid and serum in multiple sclerosis and other conditions. *Clin. Chem.* 38 2449–2454. 1458583

[B34] Nobili V. Siotto M. Bedogni G. Ravà L. Pietrobattista A. Panera N. (2013). Levels of serum ceruloplasmin associate with pediatric nonalcoholic fatty liver disease. *J. Pediatr. Gastroenterol. Nutr.* 56 370–375. 10.1097/MPG.0b013e31827aced4 23154483

[B35] Oliveira S. R. Kallaur A. P. Simão A. N. C. Morimoto H. K. Lopes J. Panis C. (2012). Oxidative stress in multiple sclerosis patients in clinical remission: association with the expanded disability status scale. *J. Neurol. Sci.* 321 49–53. 10.1016/j.jns.2012.07.045 22883481

[B36] Ortiz G. G. Macías-Islas M. A. Pacheco-Moisés F. P. Cruz-Ramos J. A. Sustersik S. Barba E. A. (2009). Oxidative stress is increased in serum from mexican patients with relapsing-remitting multiple sclerosis. *Dis. Markers* 26 35–39. 10.3233/DMA-2009-0602 19242067 PMC3833233

[B37] Ortiz G. G. Pacheco-Moisés F. P. Bitzer-Quintero O. K. Ramírez-Anguiano A. C. Flores-Alvarado L. J. Ramírez-Ramírez V. (2013). Immunology and oxidative stress in multiple sclerosis: clinical and basic approach. *Clin. Dev. Immunol.* 2013:708659. 10.1155/2013/708659 24174971 PMC3794553

[B38] Pasquali L. Pecori C. Lucchesi C. LoGerfo A. Iudice A. Siciliano G. (2015). Plasmatic oxidative stress biomarkers in multiple sclerosis: relation with clinical and demographic characteristics. *Clin. Biochem.* 48 19–23. 10.1016/j.clinbiochem.2014.09.024 25300461

[B39] Polman C. H. Reingold S. C. Banwell B. Clanet M. Cohen J. A. Filippi M. (2011). Diagnostic criteria for multiple sclerosis: 2010 revisions to the McDonald criteria. *Ann. Neurol.* 69 292–302. 10.1002/ana.22366 21387374 PMC3084507

[B40] Rice-Evans C. Miller N. (1994). Total antioxidant status in plasma and body fluids. *Methods Enzym.* 234 274–279. 10.1016/0076-6879(94)34095-1 7808295

[B41] Ristori G. Brescianini S. Pino A. Visconti A. Vittori D. Coarelli G. (2011). Serum elements and oxidative status in clinically isolated syndromes: imbalance and predictivity. *Neurology* 76 549–555. 10.1212/WNL.0b013e31820af7de 21300970

[B42] Schosinsky K. H. Lehmann H. P. Beeler M. F. (1974). Measurement of ceruloplasmin from its oxidase activity in serum by use of o-dianisidine dihydrochloride. *Clin. Chem.* 20 1556–1563. 4214636

[B43] Sheykhansari S. Kozielski K. Bill J. Sitti M. Gemmati D. Zamboni P. (2018). Redox metals homeostasis in multiple sclerosis and amyotrophic lateral sclerosis: a review review. *Cell Death Dis.* 9:348. 10.1038/s41419-018-0379-2 29497049 PMC5832817

[B44] Simó J. Joven J. Clivillé X. Sans T. (1994). Automated latex agglutination immunoassay of serum ferritin with a centrifugal analyzer. *Clin. Chem.* 40 625–629. 8149621

[B45] Singh A. Vyas V. Montani E. Cartelli D. Parazzoli D. Oldani A. (2012). Investigation of in vitro cytotoxicity of the redox state of ionic iron in neuroblastoma cells. *J. Neurosci. Rural Pract.* 3 301–310. 10.4103/0976-3147.102611 23188983 PMC3505322

[B46] Singh A. V. (2010). Multiple sclerosis takes venous route: CCSVI and liberation therapy. *Indian J. Med. Sci.* 64 337–340. 10.4103/0019-5359.99879 22918077

[B47] Singh A. V. Zamboni P. (2009). Anomalous venous blood flow and iron deposition in multiple sclerosis. *J. Cereb. Blood Flow Metab.* 29 1867–1878. 10.1038/jcbfm.2009.180 19724286

[B48] Siotto M. Aprile I. Simonelli I. Pazzaglia C. Ventriglia M. Santoro M. (2017). An exploratory study of BDNF and oxidative stress marker alterations in subacute and chronic stroke patients affected by neuropathic pain. *J. Neural Transm.* 124 1557–1566. 10.1007/s00702-017-1805-9 29086097

[B49] Siotto M. Pasqualetti P. Marano M. Squitti R. (2014). Automation of o-dianisidine assay for ceruloplasmin activity analyses: usefulness of investigation in Wilson’s disease and in hepatic encephalopathy. *J. Neural Transm.* 121 1281–1286. 10.1007/s00702-014-1196-0 24663495

[B50] Siotto M. Simonelli I. Pasqualetti P. Mariani S. Caprara D. Bucossi S. (2016). Association between serum ceruloplasmin specific activity and risk of Alzheimer’s disease. *J. Alzheimers. Dis.* 50 1181–1189. 10.3233/JAD-150611 26836154

[B51] Skikne B. S. Flowers C. H. Cook J. D. (1990). Serum transferrin receptor: a quantitative measure of tissue iron deficiency. *Blood* 75 1870–1876. 2331526

[B52] Squitti R. Ghidoni R. Simonelli I. Ivanova I. D. Colabufo N. A. Zuin M. (2018a). Copper dyshomeostasis in Wilson disease and Alzheimer’s disease as shown by serum and urine copper indicators. *J. Trace Elem. Med. Biol.* 45 181–188. 10.1016/j.jtemb.2017.11.005 29173477

[B53] Squitti R. Siotto M. Assenza G. Giannantoni N. M. Rongioletti M. Zappasodi F. (2018b). Prognostic value of serum copper for post-stroke clinical recovery: a pilot study. *Front. Neurol.* 9:333. 10.3389/fneur.2018.00333 29899723 PMC5988843

[B54] Squitti R. Salustri C. Siotto M. Ventriglia M. Vernieri F. Lupoi D. (2010). Ceruloplasmin/Transferrin ratio changes in Alzheimer’s disease. *Int. J. Alzheimers Dis.* 2011:231595. 10.4061/2011/231595 21234401 PMC3014694

[B55] Squitti R. Simonelli I. Ventriglia M. Siotto M. Pasqualetti P. Rembach A. (2014). Meta-analysis of serum non-ceruloplasmin copper in Alzheimer’s disease. *J. Alzheimers. Dis.* 38 809–822. 10.3233/JAD-131247 24072069

[B56] Stys P. K. Zamponi G. W. Van Minnen J. Geurts J. J. G. (2012). Will the real multiple sclerosis please stand up? *Nat. Rev. Neurosci.* 13 507–514. 10.1038/nrn3275 22714021

[B57] Taguchi I. Toyoda S. Takano K. Arikawa T. Kikuchi M. Ogawa M. (2013). Irbesartan, an angiotensin receptor blocker, exhibits metabolic, anti-inflammatory and antioxidative effects in patients with high-risk hypertension. *Hypertens. Res.* 36 608–613. 10.1038/hr.2013.3 23425956

[B58] Tang W. H. W. Wu Y. Hartiala J. Fan Y. Stewart A. F. R. Roberts R. (2012). Clinical and genetic association of serum ceruloplasmin with cardiovascular risk. *Arterioscler. Thromb. Vasc. Biol.* 32 516–522. 10.1161/ATVBAHA.111.237040 22075249 PMC3262121

[B59] Tasset I. Agüera E. Sánchez-López F. Feijóo M. Giraldo A. I. Cruz A. H. (2012). Peripheral oxidative stress in relapsing-remitting multiple sclerosis. *Clin. Biochem.* 45 440–444. 10.1016/j.clinbiochem.2012.01.023 22330938

[B60] Träger U. Tabrizi S. J. (2013). Peripheral inflammation in neurodegeneration. *J. Mol. Med.* 91 673–681. 10.1007/s00109-013-1026-0 23546523

[B61] Twomey P. J. Viljoen A. House I. M. Reynolds T. M. Wierzbicki A. S. (2006). Copper:caeruloplasmin ratio. *J. Clin. Pathol.* 60 441–442. 10.1136/jcp.2006.041756 17405985 PMC2001115

[B62] Vassalle C. Bianchi S. Battaglia D. Landi P. Bianchi F. Carpeggiani C. (2012). Elevated levels of oxidative stress as a prognostic predictor of major adverse cardiovascular events in patients with coronary artery disease. *J. Atheroscler. Thromb.* 19 712–717. 10.5551/jat.12740 22785135

[B63] Walshe J. M. (2003). Wilson’s disease: the importance of measuring serum caeruloplasmin non-immunologically. *Ann. Clin. Biochem.* 40 115–121. 10.1258/000456303763046021 12662398

[B64] Wolf P. (1982). Ceruloplasmin: methods and clinical use. *Crit. Rev. Clin. Lab. Sci.* 17 229–245. 10.3109/10408368209107037 6751692

[B65] Zamboni P. Galeotti R. Menegatti E. Malagoni A. M. Tacconi G. Dall’ara S. (2009). Chronic cerebrospinal venous insufficiency in patients with multiple sclerosis. *J. Neurol. Neurosurg. Psychiatry* 80 392–399. 10.1136/jnnp.2008.157164 19060024 PMC2647682

[B66] Zamboni P. Tesio L. Galimberti S. Massacesi L. Salvi F. D’Alessandro R. (2018). Efficacy and safety of extracranial vein angioplasty in multiple sclerosis: a randomized clinical trial. *JAMA Neurol.* 75 35–43. 10.1001/jamaneurol.2017.3825 29150995 PMC5833494

[B67] Zivadinov R. Schirda C. Dwyer M. G. Haacke M. E. Weinstock-Guttman B. Menegatti E. (2010). Chronic cerebrospinal venous insufficiency and iron deposition on susceptibility-weighted imaging in patients with multiple sclerosis: a pilot case-control study. *Int. Angiol.* 29 158–175. 20351672

[B68] Zivadinov R. Tavazzi E. Bergsland N. Hagemeier J. Lin F. Dwyer M. G. (2018). Brain iron at quantitative mri is associated with disability in multiple sclerosis. *Radiology* 289 487–496. 10.1148/radiol.2018180136 30015589 PMC6219694

